# Estrogen protects against liver damage in sepsis through inhibiting oxidative stress mediated activation of pyroptosis signaling pathway

**DOI:** 10.1371/journal.pone.0239659

**Published:** 2020-10-01

**Authors:** Zihan Xu, Shengzhi Mu, Xia Liao, Ronghui Fan, Wenjie Gao, Yaowen Sun, Wujun Wu, Qingan Jia

**Affiliations:** 1 Department of Burns and Plastic Surgery, Affiliated Shaanxi Provincial People’s Hospital, Northwestern Polytechnical University, Xi’an, China; 2 Department of Nutrition, First Affiliated Hospital of Xi’an Jiaotong University, Xi’an, China; 3 Department of Hepatobiliary Surgery, Affiliated Shaanxi Provincial People’s Hospital, Northwestern Polytechnical University, Xi’an, China; 4 Institute of Medical Research, Northwestern Polytechnical University, Xi'an, China; National Institutes of Health, UNITED STATES

## Abstract

Sepsis was characterized by systemic inflammatory response and multisystem organ dysfunction, refering to the activation of inflammatory and oxidative stress pathways. Estrogen has been shown to have anti-inflammatory and antioxidant effects as well as extensive organ protective role. However, whether estrogen alleviates sepsis-induced liver injury and the mechanisms involved remain unknown. Septic mice were constructed by intraperitoneal injection lipopolysaccharide, and the effect of estrogen on liver injury was investigated. Furthermore, the roles of NLRP3 inhibitor MCC950 and mitochondrial ROS specific scavenger Mito-tempo, on the liver injury were explored in septic mice. Female septic mice exhibited liver damage with increased serum AST and ALT level as well as the existence of extensive necrosis, and which was more serious in male septic mice. Moreover, Ovariectomy (OVX) aggravated sepsis-induced liver damage and activation of pyroptosis signaling pathway, which was alleviated by estrogen as evidenced by decreased serum AST, ALT level and number of infiltrating inflammatory cell, as well as protein expression related to pyroptosis. OVX aggravated mitochondrial dysfunction and liver injury in septic mice was also partly reversed by Mito-tempo and MCC950. These results demonstrated that estrogen protected against sepsis-induced liver damage through alteration of mitochondrial function and activation of inflammatory-mediated pyroptosis signaling pathway.

## Introduction

Sepsis remains the major cause of mortality in surgical intensive care units (ICU) affecting millions of patients, which is characterized by the systemic inflammatory response and multisystem organ dysfunction [1], such as lung, heart, kidney and liver. Among that, liver exerts a primary role in hypermetabolism and generates acute phase proteins during systemic inflammatory response syndrome (SIRS), and it is of crucial importance in host defense and bacteria clearance [2]. More than that, it was considered that liver was the second organ influenced in sepsis, and sepsis-associated liver injury was an important predictive marker of poor prognosis in patients with sepsis [3]. However, the mechanism of liver dysfunction was not very clear in septic mice.

Mitochondria provided energy support for liver energy metabolism, which are pivotal organelles involved in sepsis liver damage. Apart from their effect on producing energy, mitochondrial electron transport was also considered as a major source of ROS in hepatocytes. In the liver suffering from sepsis, production of detrimental mitochondrial ROS induces mitochondrial oxidative injury, which makes further various mitochondrial structural and functional changes: decreased hepatic mitochondrial membrane potential and mitochondrial biogenesis with an associated reduced oxygen consumption [4], mitochondrial swelling [5], reduced mitochondrial respiratory [6], and increased mitochondrial permeability [7]. The mitochondrial dysfunction as a result of oxidative stress contributes to sepsis-induced liver dysfunction at least in part.

Among many elements affecting cell response to inflammation-associated septic liver injury, more and more researches have stressed the fundamental role of pryoptosis. Pyroptosis was inflammatory response mediated programmed cell death, which was triggered by inflammasome in a caspase-1-dependent manner [8, 9]. In sepsis, pyroptosis ruptures the plasma membrane, which results in releases of abundant inflammatory factors, and these excessive proinflammatory cytokines in turn strengthen pyroptosis resulting in various organ injury and septic death during bacterial infections [8]. Previous study indicated that inflammasome activation and pyroptosis contributed to pseudomonas sepsis in mice, which was involved in the crosstalk between inflammasome signaling and autophagy [10]. In addition, interdiction of pyroptosis through caspase-1/-11 or gasdermin D gene deficiency induces mice resistant to endotoxin-induced sepsis [11]. However, whether pyroptosis activated by inflammasome referred to sepsis-induced liver damage was not fully elucidated.

It is widely accepted that estrogen plays protective role in multiple diseases via anti-inflammatory and antioxidant effects, such as obesity [12], gastric or colonic damage [13], cardiac protection following burn trauma [14], cognitive impairment and neurodegenerative disorders in aging [15]. Furthermore, previous study demonstrated that estrogen ameliorated sepsis-induced oxidant liver and intestines injury through regulating the release of inflammatory cytokines and suppressing tissue neutrophil infiltration in rats [16]. However, whether estrogen alleviates sepsis-induced liver injury through alteration mitochondrial function and inflammatory-mediated pyroptosis signaling pathway remains unknown.

In the present study, we explored the impact of estrogen on sepsis-induced liver injury in mouse model and examined the mitochondrial function and inflammatory- mediated pyroptosis signaling pathway in the liver tissues, in order to determine whether estrogen alleviates sepsis-induced liver injury through alteration of mitochondrial function and inflammatory-mediated pyroptosis signaling pathway.

## Materials and methods

### Animals

Four week old ICR mice were purchased from Shanghai Sippr-BK laboratory animal Co. Ltd. (Shanghai, China) and raised in a controlled environment with 23 ± 1°C and a natural light/dark cycle (morning 8:00; afternoon 8:00), and were provided with water and standard diet. Animal protocols were approved by the ethics committee on experimental animals of Shaanxi provincial people’s hospital. Intraperitoneal (i.p.) injection of pentobarbital (5mg/kg) combined with cervical spondylolisthesis was used for the sacrifice of mice after the study. Animal protocols were approved by the ethics committee on Experimental Animals of Shaanxi Provincial People’s Hospital (approval ID, SPPH-2016021).

### Experimental groups and drug treatment

In the first experiment, two genders animals were randomly assigned to 4 groups (n = 28) respectively: (1) male-control: male mice underwent vehicle; (2) male-sepsis: male mice underwent LPS (Sigma-Aldrich, MO, USA); (3) female-control: female mice underwent vehicle; (4) female-sepsis: female mice underwent LPS.

The second experiment was to explore the impact of estrogen on the liver injury in sepsis mice. The female mice were randomly assigned to 4 groups (n = 28) respectively: (1) sham-control-vehicle: control mice underwent sham operation; (2) sham-sepsis-vehicle: mice underwent LPS and sham operation; (3) OVX-sepsis-vehicle: mice underwent LPS and OVX operation; (4) OVX-sepsis-estrogen: mice underwent LPS and OVX operation as well as estrogen administration. 17β-estradiol (MCE, NJ, USA) was injected i.p. at a dose of 5mg/kg/day for 7 days after OVX operation, then LPS-induced mouse sepsis model was established.

The third experiment was to explore the impact of NLRP3 inhibitor MCC950 (MCE, NJ, USA) on the liver injury in sepsis mice. Female mice were randomly assigned to 4 groups (n = 28) respectively: (1) sham-control-vehicle: control mice underwent sham operation; (2) sham-sepsis-vehicle: mice underwent LPS and sham operation; (3) OVX-sepsis-vehicle: mice underwent LPS and OVX operation; (4) OVX-sepsis-MCC950: mice underwent LPS and OVX operation as well as MCC950 administration. MCC950 was injected i.p. at a dose of 50 mg/kg, when LPS-induced mouse sepsis model was established.

The fourth experiment was to explore the impact of Mito-tempo (MCE, NJ, USA), mitochondrial ROS specific scavenger, on the liver injury in sepsis mice. Female mice were randomly assigned to 4 groups (n = 28) respectively: (1) sham-control-vehicle: control mice underwent sham operation; (2) sham-sepsis-vehicle: mice underwent LPS and sham operation; (3) OVX-sepsis-vehicle: mice underwent LPS and OVX operation; (4) OVX-sepsis-mito-tempo: mice underwent LPS and OVX operation as well as mito-tempo administration. Mito-tempo was injected i.p. at a dose of 20 mg/kg, when LPS-induced mouse sepsis model was established.

### Contrstruction of sepsis mice and mitochondria isolation of liver tissue

For generation of the LPS-induced mouse sepsis model, ICR mice were injected intraperitoneally with LPS (5 mg/kg of body weight) dissolved in 100μl sterile saline, and the animals were euthanized 24 hours after sepsis induction. Tissue mitochondrial isolation kit (Beyotime, Haimen, China) was used for mitochondrial isolation. 50mg fresh liver tissue were cut and homogenated in 500μl reagent. Homogenate composition was repeated ten times. And after homogenized tissue centrifuged (600g/5min), the supernatant was centrifugated (11,000g/10 min, 4°C) to mitochondria and preserved in storage buffer (Beyotime, Haimen, China). Assays were performed according to the manufacturer’s instructions and conducted in quadruplicate which were described previously [17].

### Measurement of superoxide production

MitoSOX™ (Thermo fisher, Wesel, Germany) was used to detect the superoxide in mitochondria specially. Red reagent in the MitoSOX™ kit is live-cell permeant and is rapidly and selectively targeted to the mitochondria. Once in the mitochondria, red reagent is oxidized by superoxide and exhibits red fluorescence. The isolated mitochondria mentioned above was subjected to MitoSOX™ working buffer for 10 minutes (37°C, dark) and detected at 510/580 nm.

### Measurement of mitochondrial membrane potential

Mitochonrial membrane potential was measured by JC-1 (Beyome, Haimen, China) specially. The isolated mitochondria was subjected to JC-1 working buffer for 20 minutes (37°C, dark) and detected red and green fluorescent signals at 490/530nm and 488/530nm respectively (excitation/emission, light). Depolarization state in mitochondrial membrane potential was reflected by the ratio of red and green signals.

### Measurement of ATP production in mitochondria

The ATP levels in Liver tissues were detected using a commercial ATP assay kit (Beyotime, Haimen, China) according to the protocol provided by the manufacturer. In brief, the assay buffer was gently mixed with the substrate at room temperature. The mixed reagent (100 μl) was added into each well and then incubated with shaking for 15 min at room temperature. After incubation, luminescence was measured on luminescence plate reader.

### HE staining and ELISA assays

The fixed liver tissues were dehydrated in graded alcohol and then embedded in paraffin. Paraffin-embedded sections (5μm) were subjected to HE staining (including five steps: dewaxing, dyeing, dehydration, transparency and sealing) to measure the level of necrosis and infiltration of mononuclear inflammatory cells. ELISA assay kits (Cusabio Biotech, Wuhan, China) was used to measure the level of AST, ALT, IL-1β and IL-18 in serum. Assays were performed according to the manufacturer’s instructions and conducted in quadruplicate which were described previously [18].

### Western blot analysis

Mice liver tissue protein extraction and separation were performed by RIPA, which was made up of 50mM Tris (pH 7.4), 150mM NaCl, 1% NP-40, 0.5% sodium deoxycholate, 0.1% SDS, sodium orthovanadate, sodium fluoride, EDTA, leupeptin, and 10% SDS-PAGE (all purchase from Beyotime, Haimen, China) respectively. After transferred to PVDF membranes, TBST prepared 5% skim milk was used to block the membrane (room temperature 1–2 hour), which then incubated with primary antibodies against GSDMD N (1:1000), GSDMD FL (1:1000), NLRP3 (1:800), Pro-caspse1 (1:800), Caspase1 (1:800) or GAPDH (1:1000, all from Santa Cruz, CA, USA) overnight (16-24h). After incubation with secondary antibodies (1:5000, Beyotime, Haimen, China), immobilon western HRP substrate (Millipore, Eschborn, Germany) was used to detect the immune complex.

### Statistical analysis

When comparing multiple groups, one-way ANOVA was performed and when comparisons between each groups were conducted using the Student-Newman-Keuls test. IBM SPSS^®^ 20.0 for Windows was used to perform statistical analyses, with statistical significance defined as *p*<0.05.

## Results

### Female mice exhibited milder degree of liver injury, which was attributing to the protective role of estrogen in sepsis

**Fig 1:**
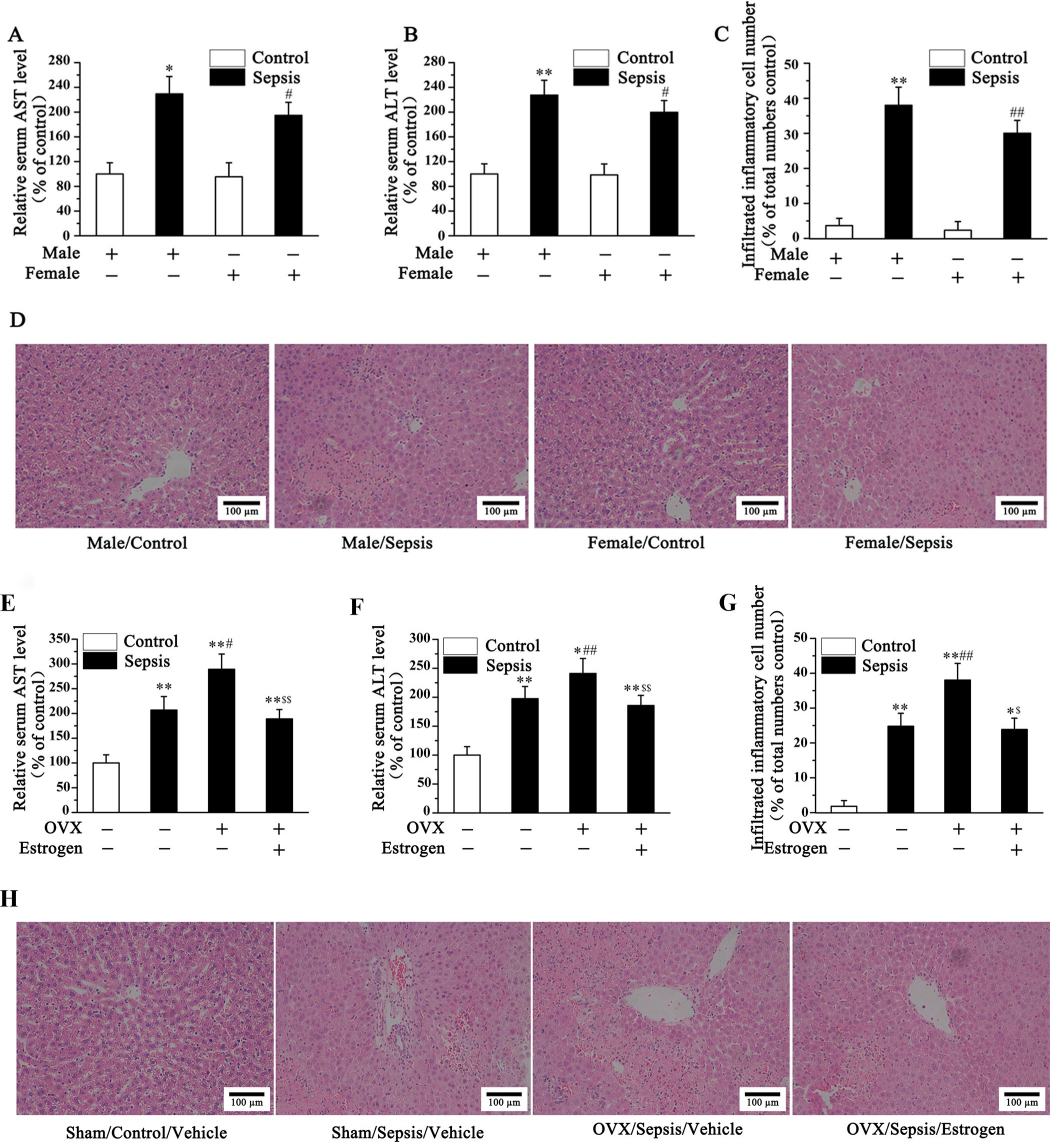
Estrogen attenuated, whereas estrogen deficiency aggravated sepsis-induced liver damage in mice (n = 28). The serum AST (A), and ALT (B) as well as the number of infiltrating inflammatory cell (C) in septic groups were significantly increased, as compared with the control groups both in male and female mice. However, the degree of elevation was lower in female mice with sepsis than that in male mice. HE staining of liver tissue also indicated the increased inflammatory cell infiltration and necrosis in septic groups (D). Estrogen deficiency in OVX mice could obviously aggravate sepsis-induced liver injury as evidenced by increased serum AST (E) and ALT (F) level as well as increased liver necrosis area (H) along with infiltration of inflammatory cell number (G), and which were mitigated after estrogen administration. (Fig A-D *P<0.05, **P <0.01 *vs*. Male/Control; ^#^*P<*0.05, ^##^*P<*0.01 *vs*. Male/Sepsis; Fig E-H *P<0.05, **P <0.01 *vs*. Sham-Vehicle; ^#^*P<*0.05, ^##^*P<*0.01 *vs*. Sham-Sepsis; ^$^*P<*0.05, ^$ $^*P<*0.01 *vs*. OVX-Sepsis).

Accumulating number of evidences has demonstrated gender differences in morbidity and mortality in patients with severe sepsis or septic shock [19, 20]. In this part, we verified that the serum AST and ALT level as well as liver necrosis area along with the number of infiltrating inflammatory cell in septic groups were significantly increased, as compared with the control groups both in male and female mice. However, the degree of elevation of AST and ALT, as well as necrosis area along with the number of infiltrating inflammatory cell were lower in female mice with sepsis than in male mice. There was no difference between the control group of different genders in the level of AST and ALT, as well as necrosis area along with the number of infiltrating inflammatory cell (Fig 1A–1D).

We then explored the impact of estrogen on sepsis-induced liver injury. As shown in this part, estrogen deficiency in OVX mice could obviously aggravated sepsis-induced liver injury as evidenced by increased serum AST and ALT level as well as increased liver necrosis area along with infiltration of inflammatory cell number. And the mitigated liver injury with decreased serum AST and ALT level as well as increased liver necrosis area along with infiltration of inflammatory cell number were proved after estrogen administration (Fig 1E–1H). In this section, we confirmed that estrogen deficiency aggravates sepsis-induced liver damage, and in septic female mice or OVX mice with estrogen administration exhibited milder degree of liver injury.

### NLRP3-mediated pyroptosis signaling pathway was activated in the liver of female septic mice

**Fig 2:**
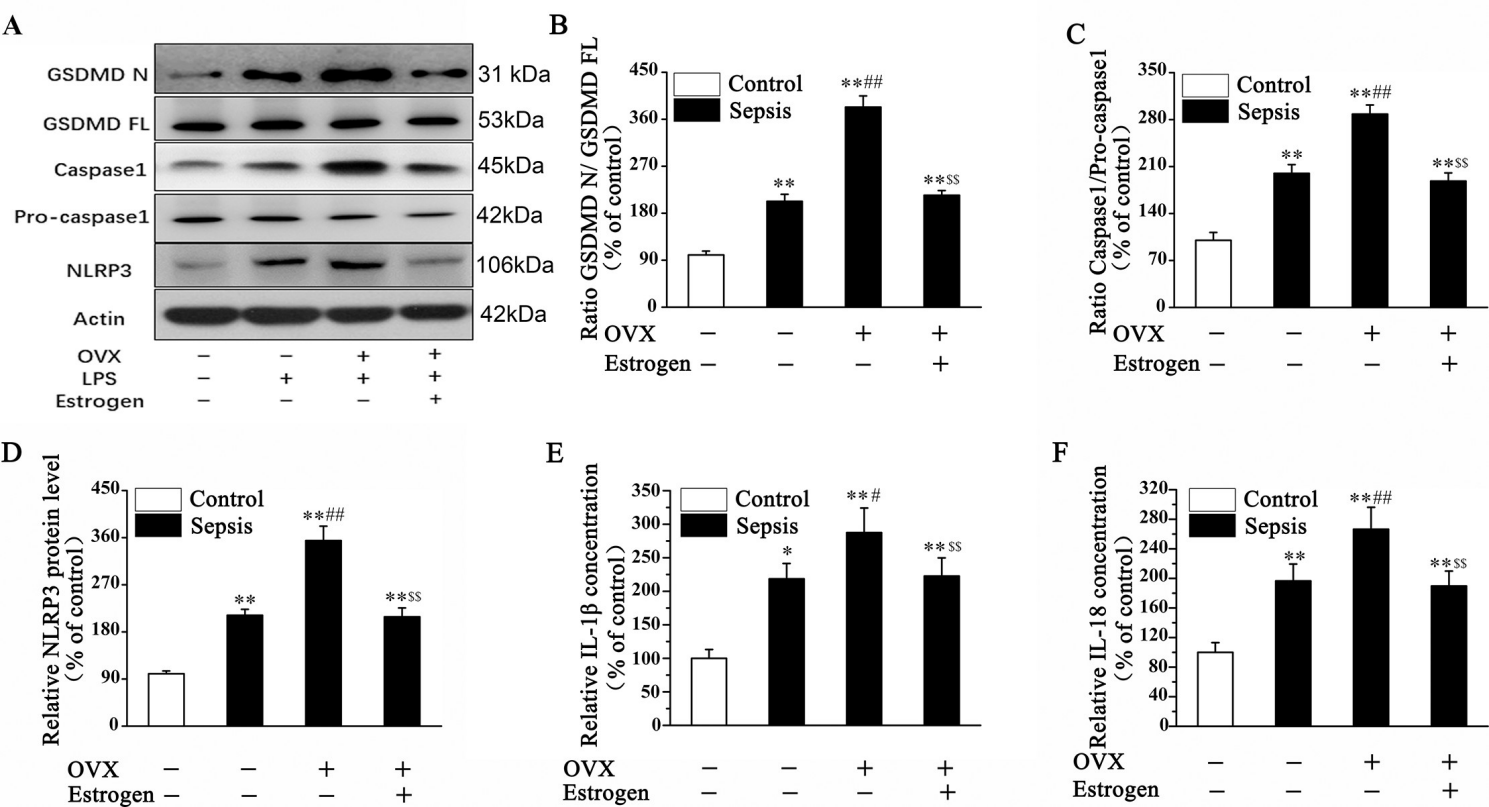
NLRP3-mediated pyroptosis signaling pathway was activated in the liver of female septic mice. The protein levels of GSDMD N, GSDMD FL, Caspase1, Pro-caspase1 and NLRP3 in liver tissue were measured in female septic mice by WB, and estrogen administration could inhibit the expression of NLRP3, as well as cleaved GSDMD and Caspase-1. (Fig 2A and 2D). The ratios of GSDMD N/ GSDMD FL and Caspase 1/ Pro caspase 1 were increased in the liver of female septic mice, which were further increased in OVX mice. And estrogen administration could reverse the increased ratios (Fig 2B and 2C). The serum level of IL-1β and IL-18 were examined by using ELISA assays, and which were increased in female septic mice. OVX induced further increase in serum level of IL-1β (E) and IL-18 (F) in septic mice could be abolished by estrogen administration. (*P<0.05, **P <0.01 *vs*. Sham-Vehicle; ^#^*P<*0.05, ^##^*P<*0.01 *vs*. Sham-Sepsis; ^$^*P<*0.05, ^$ $^*P<*0.01 *vs*. OVX-Sepsis).

Increasingly researches indicated the important role of NLRP3-mediated the activation of pyroptosis signaling pathway in liver injury [21, 22]. So we examined expression of proteins related to NLRP3-mediated pyroptosis signal pathway in the liver of female septic mice subjected to estrogen administration to elucidate the protective of estrogen against septic liver injury. As shown in this part, protein level of cleaved GSDMD and caspase-1 as well as NLRP3 in the liver of LPS treated mice were significantly increased, which were further increased in OVX mice. And it was found that estrogen administration could dramatically reverse sepsis-induced up-regulation of cleaved GSDMD and caspase-1 as well as NLRP3 (Fig 2A and 2D). The ratios of GSDMD N/ GSDMD FL and caspase 1/ Pro-caspase 1 were increased in the liver of female septic mice, and which were further increased in OVX mice. While the decreased ratios were proved after estrogen administration in OVX mice with sepsis (Fig 2B and 2C). Moreover, that OVX induced further increase in serum level of IL-1β (Fig 2E) and IL-18 in septic mice could be abolished by estrogen administration (Fig 2F). In this section, we confirmed estrogen protects the liver against sepsis-induced damage in mice relating to the suppression of NLRP3 and associated pyroptosis signaling pathway.

### The protective estrogen against sepsis-induced liver damage in mice was relating to NLRP3-mediated activation of pyroptosis signaling pathway

**Fig 3:**
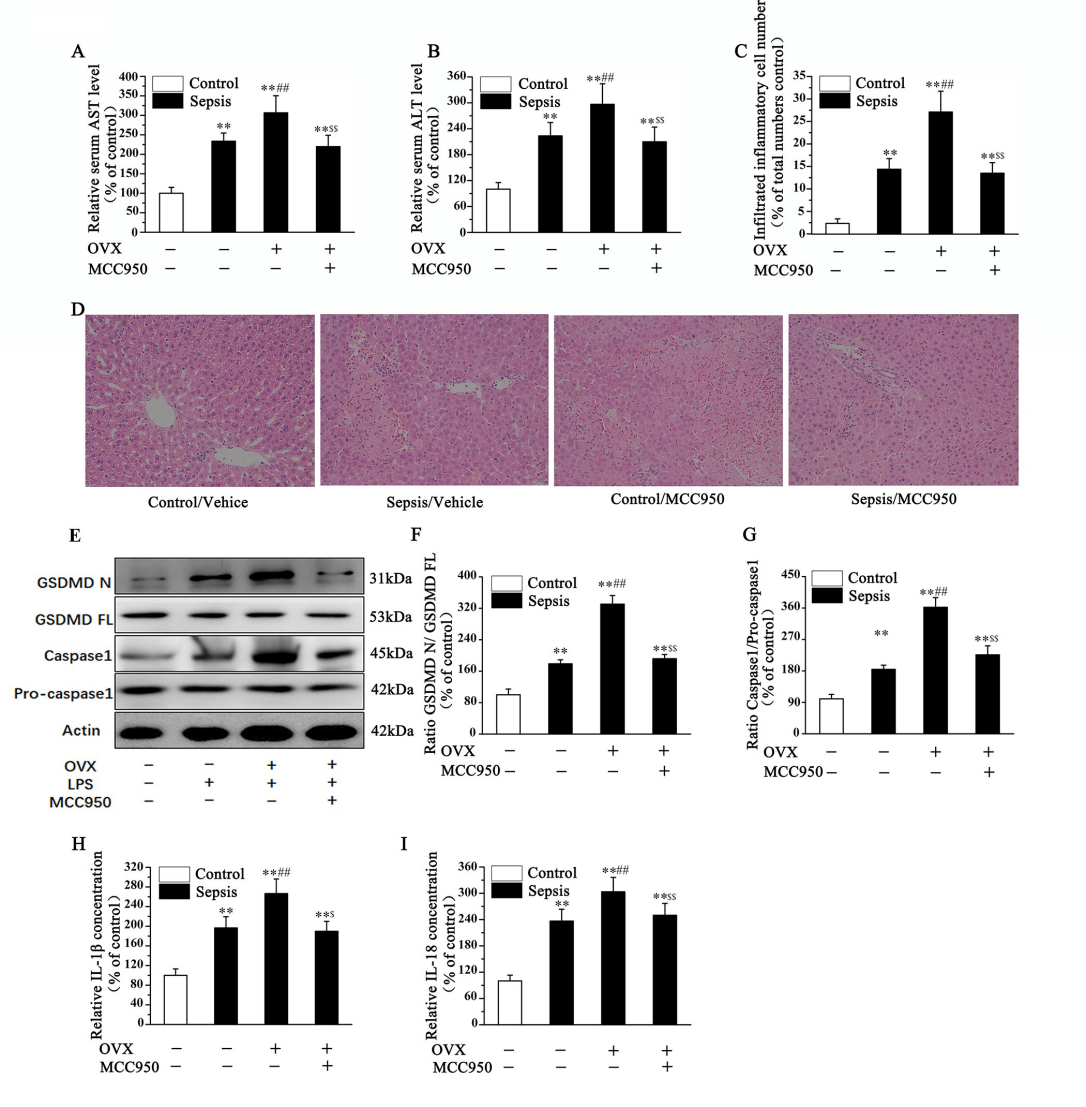
Sepsis-induced liver damage in mice was relating to NLRP3-mediated activation of pyroptosis signaling pathway. The serum levels of AST (A), ALT (B), inflammatory cell infiltration (C), and necrosis (D) in septic mice were significantly increased as compared with the control groups, and levels were further increased in OVX mice and reversed after MCC950 administration. The protein level of GSDMD N, GSDMD FL, Caspase-1, and Pro-caspase1 (E-G) in liver tissue were measured, and the expression of cleaved GSDMD and caspase-1 were increased after OVX, and which were inhibited by MCC950 administration. The ratios of GSDMD N/ GSDMD FL and Caspase 1/ Pro caspase 1 were increased in the liver of female septic mice, which were further increased in OVX mice. And MCC950 administration could reverse the increased ratios (Fig 3F and 3G). The increased serum level of IL-1β (H) and IL-18 (I) in OVX mice were also abolished by MCC950 administration. (*P<0.05, **P <0.01 *vs*. Sham-Vehicle; ^#^*P<*0.05, ^##^*P<*0.01 *vs*. Sham-Sepsis; ^$^*P<*0.05, ^$ $^*P<*0.01 *vs*. OVX-Sepsis).

To further investigate whether NLRP3-mediate pyroptosis was involved in the protection of estrogen on the liver dysfunction in septic female mice, we use MCC950, a inhibitor of NLRP3, to treat OVX mice. As shown in this part, OVX caused further increase in serum level of AST and ALT, as well as the number of infiltrating inflammatory cell and along with liver necrosis area in the liver of sepsis mice could also be reversed by MCC950 administration (Fig 3A–3D). In addition, protein level of cleaved GSDMD and caspase-1 in the liver of LPS treated mice were significantly increased. And OVX induced further increase protein expression related to pyroptosis was abolished after MCC950 administration (Fig 3E). The ratios of GSDMD N/ GSDMD FL and caspase 1/ Pro-caspase 1 were increased in OVX mice. And the decreased ratios were proved after MCC950 administration (Fig 3F and 3G). Moreover, that OVX induced further increase in serum level of IL-1β and IL-18 in septic mice could be abolished by MCC950 administration (Fig 3I). In this section, we confirmed that the protective estrogen against sepsis-induced liver damage in mice was through suppressing NLRP3-mediated the activation of pyroptosis signaling pathway.

### Estrogen protects the liver in septic mice through suppressing ROS-mediated NLRP3 activation and mitochondrial dysfunction

**Fig 4:**
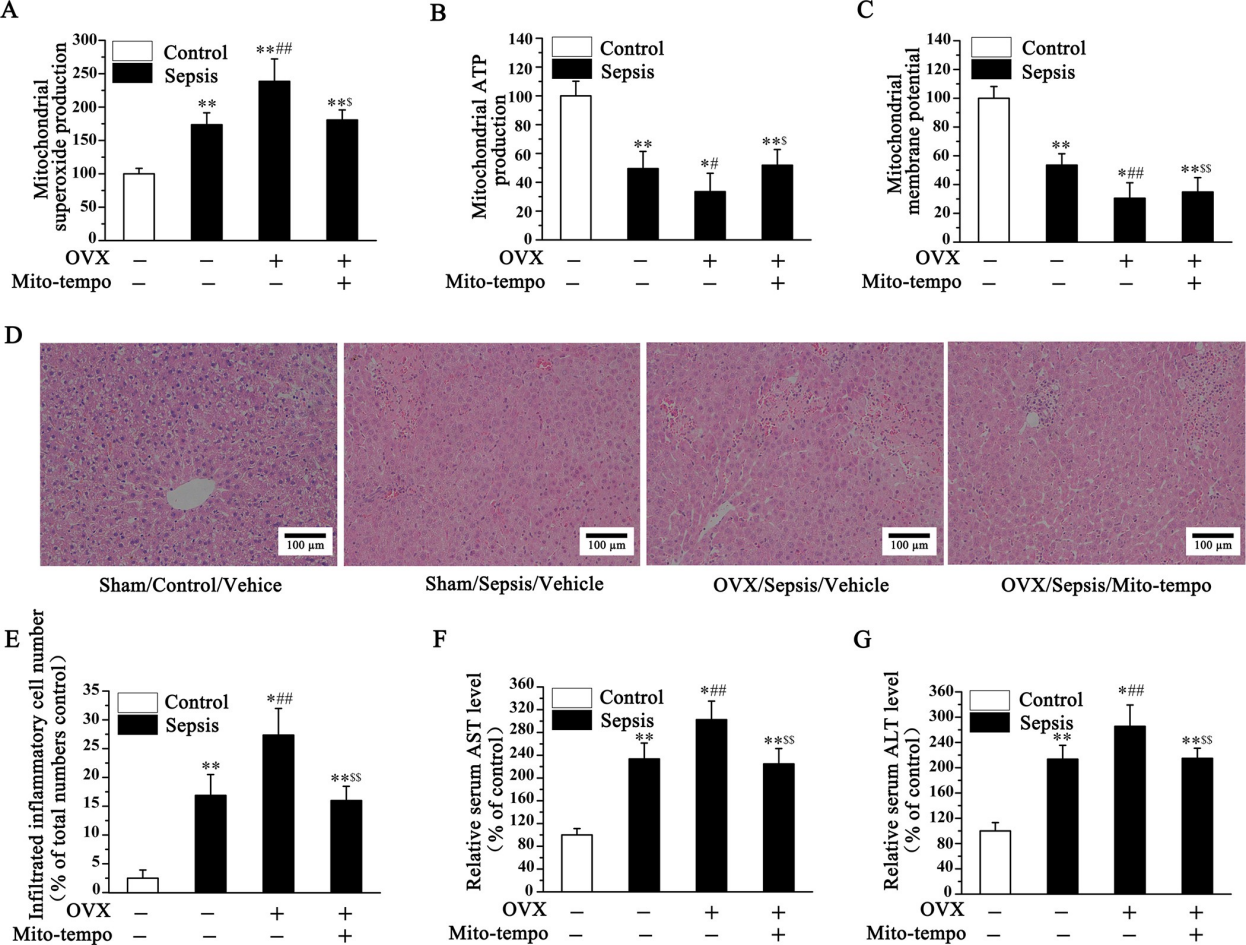
Estrogen protected the liver in septic mice through suppressing ROS-mediated NLRP3 activation and mitochondrial dysfunction. The mitochondrial function was measured by mitochondrial superoxide production (A), mitochondrial ATP production (B) and mitochondrial membrane potential (C), and which was increased in LPS treatment group, aggravated in OVX group, and inhibited by Mito-tempo administration. And the tissue necrosis and inflammatory cell infiltration (D, E), as well as the serum AST (F) and ALT (G) in septic male mice were all significantly increased in LPS treatment group, aggravated in OVX group, and inhibited by Mito-tempo administration. (*P<0.05, **P <0.01 *vs*. Sham-Vehicle; ^#^*P<*0.05, ^##^*P<*0.01 *vs*. Sham-Sepsis; ^$^*P<*0.05, ^$ $^*P<*0.01 *vs*. OVX-Sepsis).

As we all known that NLRP3 activation was largely attributed to excessive ROS generation [23, 24], which mainly came from the electron transport chain of mitochondria [25]. So, we examined mitochondrial function and ROS level in the liver of sepsis mice. As shown in this part, LPS treatment could induce mitochondrial dysfunction in the liver, as evidenced by increased mitochondrial superoxide production, decreased mitochondrial ATP production and membrane potential, as compared with the control mice, which was aggravated by OVX. However, the damaged mitochondria could be rescued by Mito-tempo, mitochondrial ROS specific scavenger, which indicating estrogen protected against liver damage via suppressing mitochondrial ROS generation (Fig 4A–4C). What is more, it was also proved that Mito-tempo could reverse OVX-mediated in number of infiltrating inflammatory cell in liver, as well as serum level of AST and ALT in sepsis mice (Fig 4D–4F). These results indicated that the protective of estrogen against sepsis-induced liver damage was partly attributed to excessive ROS generation mediated mitochondrial dysfunction.

## Discussion

Estrogen has been considered as a medical tool to counteract inflammation and clinical symptoms in multiple disorders including acute spinal cord injury [26], multiple sclerosis [27], neurovascular/neuroimmune disease [28], chronic spinal cord injury [29], severe burn injury [30], chronic liver injury [31], inflammatory bowel disease [31], aging [32] and obesity [12]. Inflammation was involved in the development of sepsis-associated diseases and contributed to the mortality of the patients with severe sepsis. Previous studies indicated that estrogen could attenuate sepsis-related disorder through reducing hypothalamic inflammation [33]. In recent years, accumulating number of researches has highlighted the role of inflammation-mediated pyroptosis in the development of sepsis. What is more, previous studies indicated that NLRP3 inflammasome deficiency or suppression protects against sepsis related injury via suppressing inflammation induced caspase-7 cleavage and pyroptosis or augmenting PKA signaling pathway [34]. Chen et al. demonstrated that sepsis-induced acute liver damage was inhibited via depressing hepatic cells pyroptosis [9]. Consistent with these results in this study, NLRP3 inflammasome mediated pyroptosis contributed to the progression of sepsis and its inhibition could mitigate sepsis-induced liver injury by estrogen administration as shown by decreases in serum AST and ALT level in septic mice.

A great number of researches indicated that mitochondrial oxidative stress was implicated in a variety of diseases and estrogen exerted protective role through alleviating mitochondrial oxidative stress induced mitochondrial dysfunction in multiple diseases, such as vascular disease [35], neurological disease [36], Alzheimer's disease [37]. Moreover, Sener et al. demonstrated that estrogen mitigated the liver and intestines injury in septic rat, which might be attributed to its antioxidant properties [16]. Zang et al. showed that targeting inhibition of mitochondrial oxidative stress could enhance cardiac function in sepsis model [38]. Estrogen is a sex hormone which is involved in various physiological and pathophysiological distinction between the two genders [39]. Taking into account the gender/sex differences will open a new arena in sepsis therapy. In the present study, we provided for the first experimental evidence that estrogen significantly alleviate sepsis-induced liver injury through blocking mitochondrial oxidative stress mediated mitochondrial dysfunction as evidenced by decreased mitochondrial superoxide production, increased mitochondrial ATP production and mitochondrial membrane potential.

Notably, mitochondria-targeted vitamin E, which targeted inhibit mitochondrial oxidative stress, improved cardiac function through alleviating tissue-level inflammation during sepsis [38]. More interestingly, time course research demonstrated that mitochondrial dysfunction occurred earlier than appearance of inflammatory responses in the heart, indicating that the changes in mitochondrial function might result in induction of myocardial inflammation during sepsis. In the present study, we demonstrated that Mito-tempo mitigated liver damage in septic mice which was ascribed suppression NLRP3-mediated the activation of pyroptosis signal pathway in septic mice liver.

From our experimental results, we found that estrogen could mitigate sepsis-induced liver injury, as evidenced by decreased serum AST and ALT level, as well as improved mitochondrial dysfunction and activation of pyroptosis signaling pathway, along with decreased superoxide production in mitochondria and decreased protein expression of pyroptosis relative protein in the septic liver. And this study demonstrated estrogen alleviates sepsis-induced liver injury through alteration of mitochondrial function and inflammatory-mediated pyroptosis signaling pathway.

## Supporting information

S1 Fig (PPTX)
