# Supplementary material for: Estrogen protects against liver damage in sepsis through inhibiting oxidative stress mediated activation of pyroptosis signaling pathway
Source: PLoS One. 2020 Oct 1;15(10):e0239659. doi: 10.1371/journal.pone.0239659 (PMC7529240; doi:10.1371/journal.pone.0239659)
Supplement: S1 Fig — (PPTX) [file pone.0239659.s001.pptx]

## Slide 1
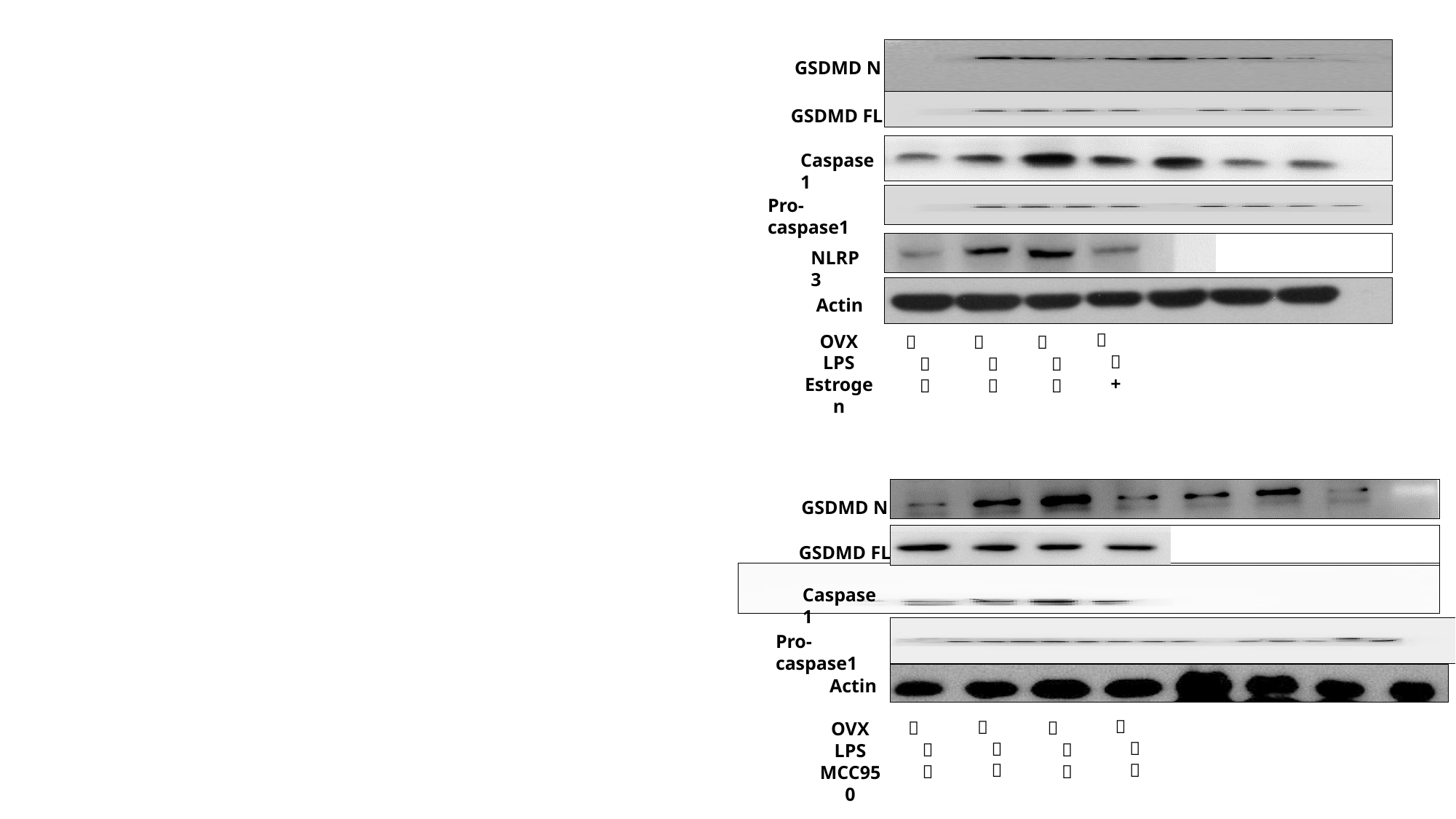

GSDMD N
GSDMD FL
Caspase1
Pro-caspase1
NLRP3
Actin
＋
＋
+
OVX
LPS
Estrogen
－
－
－
－
＋
－
＋
＋
－
GSDMD N
GSDMD FL
Caspase1
Pro-caspase1
Actin
＋
＋
＋
－
＋
－
－
－
－
＋
＋
－
OVX
LPS
MCC950
